# Bridging the gap in precision medicine: TranSYS training programme for next-generation scientists

**DOI:** 10.3389/fmed.2024.1348148

**Published:** 2024-05-24

**Authors:** Lara Andreoli, Catalina Berca, Sonja Katz, Maryna Korshevniuk, Ritchie M. Head, Kristel Van Steen, TranSYS Consortium, Kristel Van Steen

**Affiliations:** ^1^Department of Public Health and Primary Care, Centre for Biomedical Ethics and Law, KU Leuven, Leuven, Belgium; ^2^Epithelial Carcinogenesis Group, Molecular Oncology Programme, Spanish National Cancer Research Centre (CNIO), Madrid, Spain; ^3^Laboratory of Systems and Synthetic Biology, Wageningen University and Research, Wageningen, Netherlands; ^4^LifeGlimmer GmbH, Berlin, Germany; ^5^Department of Radiology and Nuclear Medicine, Erasmus MC, Rotterdam, Netherlands; ^6^Genetics Department, University Medical Center Groningen, Groningen, Netherlands; ^7^Ceratium BV, Amsterdam, Netherlands; ^8^Laboratory for Systems Genetics, GIGA-R Medical Genomics, University of Liège, Liège, Belgium; ^9^Laboratory for Systems Medicine, Department of Human Genetics, KU Leuven, Leuven, Belgium

**Keywords:** innovative training network, interdisciplinary, precision medicine, artificial intelligence, omics, training, Marie Skłodowska-Curie Actions

## Abstract

**Introduction:**

In the evolving healthcare landscape, precision medicine's rise necessitates adaptable doctoral training. The European Union has recognized this and promotes the development of international, training-focused programmes called Innovative Training Networks (ITNs). In this article, we introduce TranSYS, an ITN focused on educating the next generation of precision medicine researchers. In an ambition to go beyond describing the consortium goals, our article explores two key aspects of ITNs: the training and collaboration.

**Methods:**

Using self-report questionnaires, we evaluate the scientific, professional, and personal growth of ESRs over the duration of the ITN and investigate whether this can be linked to network activities.

**Results:**

Our quantitative analysis approach reveals substantial improvements in scientific, professional, and social skills among young researchers facilitated by the engagement in this interdisciplinary network. We provide case studies underlining the advantages of collaborative environments, featuring innovative scientific exchange within TranSYS.

**Discussion:**

While challenging, ITNs foster positive growth in young researchers, yet exhibit weaknesses such as balancing stakeholder interests and partner commitment. We believe this study may benefit a variety of stakeholders, from prospective ITN creators to industry partners, to design better sustainable training networks going forward.

## 1 Introduction

Precision medicine (PM) represents an evolving field within the healthcare sector, centered on the fundamental premise that an individual's reaction to diseases and therapeutic interventions is intricately shaped by their unique genetic makeup, environment, and a constellation of personalized biological factors. By meeting the specific needs and characteristics of each patient, PM intends to make both clinical decision-making and data communication more effective and to minimize potential side effects (1), and also improve patients compliance. The ambition is to use individuals' phenotypes and genotypes (e.g., molecular profiling, biomarkers, lifestyle data) to tailor the right disease prevention or therapeutic strategies for the right person at the right time. To approach the challenges associated with this paradigm-shift in disease prevention and healthcare, the involvement and collaboration of key stakeholders, including healthcare professionals, academia, policy makers, industry, and patients is required. In order to achieve this goal, understanding the molecular-level causality of pathogenesis becomes crucial. Highly interdisciplinary skill sets are now needed to exploit big datasets and scientific advances that can drive improvements in the prevention, diagnosis, and development of tailor-made interventions for individuals or groups of individuals.

The collaborative nature of precision medicine, which integrates expertise from diverse fields such as genomics, informatics, and clinical practice, demands a commitment to ethical standards. Transparent communication across disciplines guarantees a shared understanding of goals, methodologies, and potential implications, fostering a cohesive and responsible research environment. Ethical considerations extend to acknowledging and addressing biases, promoting inclusivity, and safeguarding against the misuse of data. Socially, a commitment to interdisciplinarity promotes a holistic approach to healthcare that reflects the complex interplay of genetic, environmental, and lifestyle factors. In this way, precision medicine can fulfill its promise of providing tailored, effective, and ethically sound healthcare solutions for individuals and communities.

In this context, Innovative Training Networks (ITNs), and under the EU's Horizon Europe programme the new Doctoral Networks have emerged as valuable frameworks for global research and education collaborations. ITNs are an example of a multinational interdisciplinary PhD programme jointly implemented by academic institutions, industries, and others across Europe (2). These training networks are designed to facilitate transnational cooperation, fostering the exchange of expertise, ideas, and best practices among researchers, and professionals worldwide. Their origin can be traced back to the late 20^*th*^ century, a period marked by increasing globalization and the rapid evolution of information and communication technologies. In response to the need for enhanced international collaboration, the European Commission took a pioneering step in 1990 by launching the Marie Skłodowska-Curie Actions (MSCA) program, which aimed to support mobility and training for researchers within Europe (3, 4). ITNs revolve around several key focuses, each contributing to the enrichment of research, education, and professional development on a global scale (5):

- Interdisciplinary research
- Mobility and knowledge exchange
- Skill development and training
- Collaborations and consortia building
- Innovation
- Human capital development
- International cooperation

The European Union recognized the importance of developing a skilled workforce that could address current and future challenges and drive innovation in biomedicine. To meet this training need the TranSYS ITN was established to offer Early Stage Researchers (ESRs) the opportunity to gain interdisciplinary training, engage in collaborative research projects, and develop novel techniques and tools with the goal to advance PM (6). By supporting this interdisciplinary training network, the EU aims to foster innovation, promote scientific excellence, and address critical health-related issues in an increasingly interconnected world. It has evolved from joint workshops and consultations, identifying training gaps in European levels. Preliminary results from implementing pharmacogenomics in clinical settings (cardiology, oncology, and psychiatry) show potential to improve drug use, minimize adverse reactions, and reduce healthcare costs (7, 8).

The consortium at the core of TranSYS consists of a combination of expertise in various fields including Systems Medicine, Functional Genomics, Bioinformatics, Biostatistics, Integrative Biology, Artificial Intelligence (A.I.), Software Development, Blockchain, Ethics, and Pharmacogenomics, completed by industry partners at the forefront of innovative future healthcare. All ESR research projects were complemented by training covering technical (genomics, bioinformatics, health informatics, statistics, data mining, systems medicine, and ethics) and key soft skills relevant to high-level research career paths as future leaders for the Precision Medicine revolution. The ESR projects in TranSYS aim to address barriers related to translational systemics (e.g., limited access to validated data and lack of standards for data storage and curation, along with data privacy concerns) and deliver key innovations to enhance disease detection, diagnosis, treatment, and preventive healthcare.

This paper provides an in-depth exploration of the TranSYS ITN, including its inception, primary areas of focus, and notable achievements. In an ambition to go beyond simply describing goals of the consortium, we aim on giving a detailed insight into two key features of ITNs: the ESR training and collaboration. Using self-reported metrics we evaluate the scientific, professional, and personal growth of ESRs over the duration of the ITN and investigate whether this can be linked to network activities. Furthermore, we measure the scientific output of the consortium and highlight individual projects that could only be facilitated by the collaborative nature of an ITN. Finally, we provide an ESR-perspective on open challenges within ITNs and suggest possible solutions to further improve the experience for early career researchers joining a MSCA-ITN.

## 2 Results

### 2.1 Overview of ESR projects and expertise

**Figure 1:**
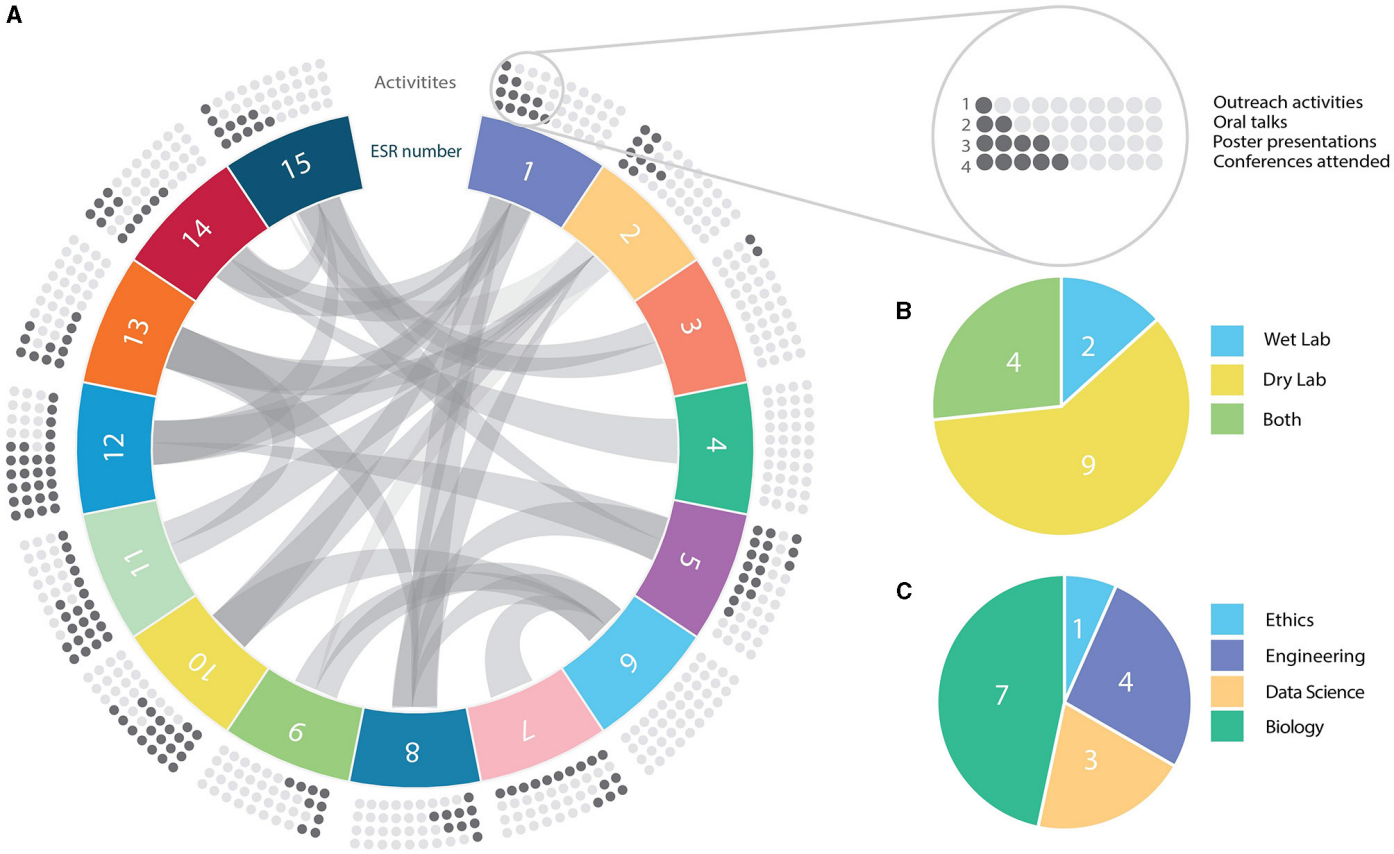
Summary on TranSYS ESRs. **(A)** Circos diagram displaying the number (panel), secondments/collaborations (chords), and the scientific output (dots) of each TranSYS ESR. **(B)** Technical nature of projects. **(C)** Combined educational background or formal training of ESRs. ESR color coding is consistent throughout the manuscript.

The ESRs recruited for TranSYS are as multidisciplinary and diverse as the program itself. Taken together, the ESRs showed a high degree of interaction within the network (Figure 1A, chords), but also with the scientific community outside TranSYS (Figure 1A, dots). The healthy mix of wet and dry lab projects (Figure 1B) enabled the onboarding of ESRs from a broad variety of backgrounds: from physics, engineering, and statistics, to genetics, molecular biology, pharmacology, and ethics (Figure 1C). A more in-depth overview of individual projects and how they fit into the frame of TranSYS work packages (WP) can be found in section *Mission and structure of the TranSYS consortium* and Table 1.

**Table 1:** Overview of ESR projects.

**ESR**	**Affiliation**	**Title of the project**	**WP**	**Lab**	**Keywords**
1	KU Leuven, Belgium	Development of individual-specific molecular networks	2	Dry	gene-based networks, multi-omics
2	KU Leuven, Belgium	Hunting for patient subtypes through image-based phenotypes as biomarkers for major gene effects in medical disorders	2	Dry	genomic data, patient stratification
3	Erasmus MC, Netherlands	GDPR regulation in translational medicine	1,2,3	Both	multi-omics, patient stratification
4	KU Leuven, Belgium	Polygenic risk score(s) in the clinic: ethical challenges and stakeholders' perspectives	2	Dry	bioethics, PRS, ELSI
5	University of Ljubljana, Faculty of Medicine, Slovenia	Personalized molecular signatures for modulating progression of metabolism associated liver disease (MAFLD) to hepatocellular carcinoma	1	Both	genome-scale metabolic models,
6	Université du Luxembourg, Luxembourg	Dissecting cellular heterogeneity of Parkinson's disease (PD) related iPS cells during aging by integrated single cell transcriptomics and imaging analysis to identify disease modifiers	1	Wet	PD, scRNA-seq, neurodegeneration
7	Spanish National Cancer Research Centre (CNIO), Spain	Personalized approaches to modulate tumor behavior using vitamin D3	1	Wet	bladder cancer, targeted therapies, patient derived organoid
8	Institut Pasteur, France	Integrated modeling of systemic autoimmune diseases	2	Dry	transcriptomics, inter-individual variability
9	Barcelona Supercomputing Center, Spain	Patient-centric data integration framework for highly dimensional data	2	Dry	data integration, system-level analysis
10	KU Leuven, Belgium	Development of a Federated Blockchain-based Clinical Architecture for Empowering Data Interoperability	1,2,3	Dry	data interoperability, data sharing
11	Max Planck Institute of Psychiatry, Germany	Identification of biological subtypes related to treatment resistant depression	2	Dry	depression, patient subtyping
12	University Medical Centre Groningen, The Netherlands	Multi-omics analysis to delineate drug-response pathways	3	Both	multi-omics, data integration, single-cell analysis
13	Max Planck Institute of Psychiatry, Germany	Understanding stress-responsive molecular networks	3	Dry	psychiatry, PRS
14	The Golden Helix Foundation, United Kingdom	Standardization of disease and population-specific genotyping panel for preemptive pharmacogenomics	3	Both	preemptive pharmacogenomics
15	LifeGlimmer GmbH, Germany	Developing data mining and A.I. tools to better understand patient heterogeneity	3	Dry	A.I., patient stratification

Displayed are the ESR number (ESR), Affiliation, Project title, Associated work package (WP), Technical nature of the project (wet lab, dry lab, both), and Keywords outlining the field of research.

### 2.2 Professional, scientific, and personal growth of ESRs throughout TranSYS

All ESR individual research projects were complemented by training covering technical (genomics, bioinformatics, health informatics, statistics, data mining, systems medicine, and ethics) and key soft skills essential for taking a leading position in the Precision Medicine of the future. With the goal of gaining a more quantitative impression on the achievements of TranSYS in terms of scientific innovation and impact of ESR training, we conducted a survey amongst ESRs. This survey included the questions regarding formal education, a variety of technical, managerial, and soft skills, as well as ratings of official TranSYS events, such as summer schools and secondments, assessing their perceived impact on the development respective skills.

**Figure 2:**
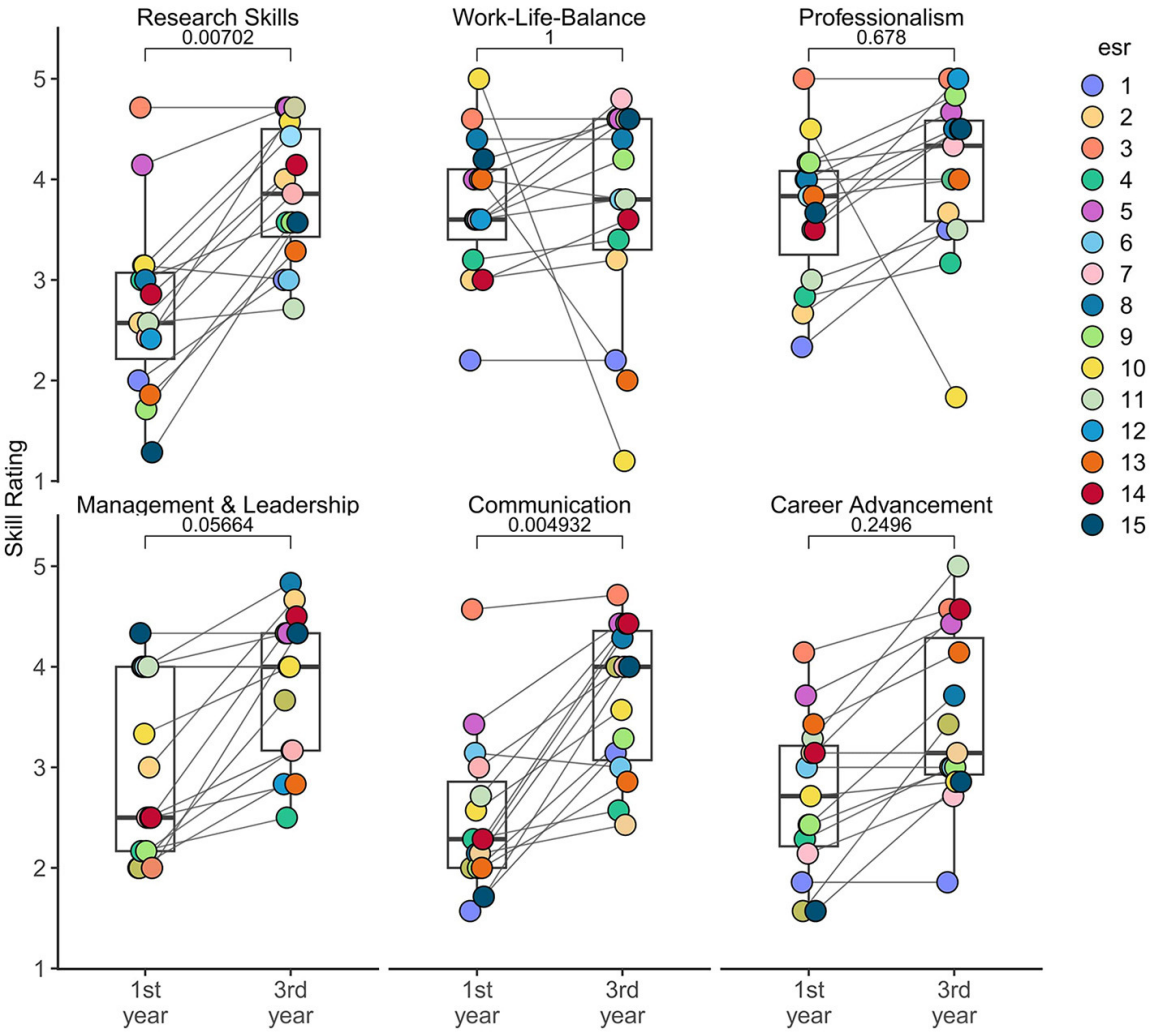
Improvement of ESRs throughout the period of TranSYS in six different major skill categories. Data was collected using a self-assessment survey upon joining TranSYS (1st year) and prior to rotating off (3rd year) (see Section 5). Each dot represents the one ESR, the lines connecting dots represent the relative change in skill rating. Boxplots indicate the summary of all ESRs in respective category and timepoint. *P*-values were derived using a Wilcoxon signed-rank test and multiple testing corrected using Bonferroni correction. A value < 0.05 can be considered significant.

To assess the development of ESRs throughout the period of TranSYS, we compared the self-reported skill ratings of a set of diverse categories from the start (1st year) to the end of the ITN period (3rd year) (Figure 2). A more detailed analysis of each category can be found in Supplementary Figure 1. It is evident that while ESRs strengthened their skills overall, especially their competences in communication (adjusted *p*-value < 0.05) and research (adjusted *p*-value < 0.01) significantly improved. Communication skills include public speaking, writing, and presentation skills, while research skills encompass areas such as knowledge of the field, critical reading, or understanding data ownership. Other categories, however, do not reflect this trend, with individuals reporting perceived indifference or even worsening of skills. Foremost, we find a lack of growth in the area of Work-Life-Balance, revolving around topics such as quality of life, healthy time management, or maintaining personal motivation. ESRs also felt like they did not grow a lot in their professionalism, referring to their competence in upholding deadlines, seeking advice, or contributing to a team.

**Figure 3:**
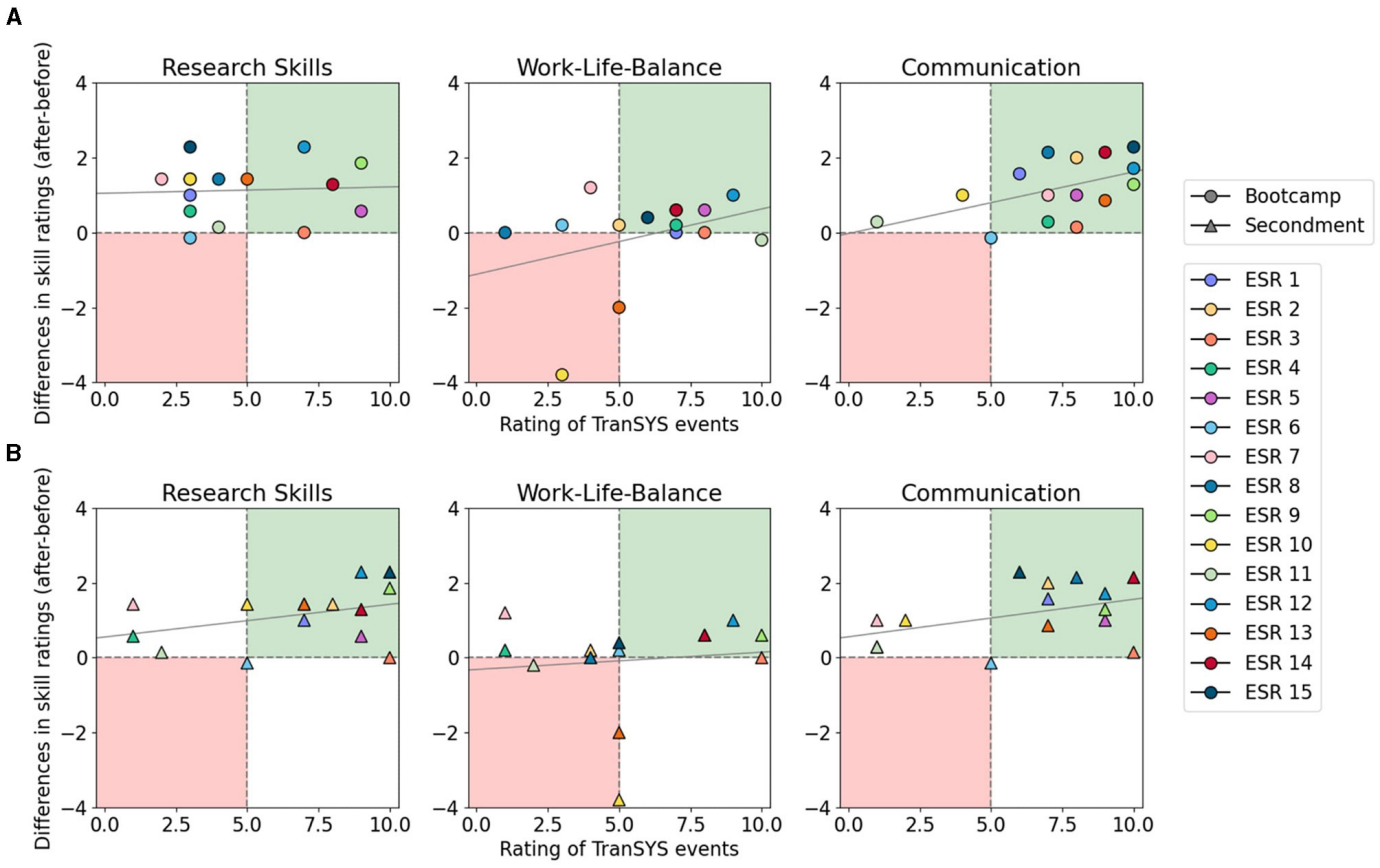
Impact of TranSYS bootcamps **(A)** and secondments **(B)** on ESR growth. The x-axis shows ESRs' perceived impact of TranSYS events on the evolution of skills. Skill improvements (y-axis) were derived by calculating the difference in subjective skill ratings of the 3rd and 1st year within TranSYS. Individuals in the upper right quadrant (green) show a positive association between event rating and skill improvement. Individuals in the lower left quadrant (red) display dissatisfaction with TranSYS events paired with a lack of growth in respective skill.

One can argue that during three years spent working in academia an improvement of skills at every level will occur regardless of the possibilities offered within an ITN. To test this, we correlated the growth of each ESR—measured as the difference between skill levels reported in the 3^*rd*^ and 1^*st*^ year—with their rating on the perceived value of TranSYS events such as summer schools (Figure 3A) and secondments (Figure 3B) for each skill category. While research skills seem to have improved independently of summer school activities (Figure 3A, left panel), TranSYS events can be clearly correlated with an improvement in communication (right), evident by the large number of ESRs in the green quadrant. Interestingly, although many valued events for their positive impact on Work-Life-Balance, no real improvement of skill was visible (middle); on the contrary, all ESRs reporting a worsening in this category were dissatisfied with the events. What summer schools were lacking in terms of impact on research skills, the secondments clearly compensated for (Figure 3B, left panel). A majority of ESRs rated their secondments very positively, going hand in hand with noticeable learning effects. A similar effect was observed for the categories communication (right), as well as management, and career advancement (Supplementary Figure 2). However, secondments were also perceived to have a negative impact across a majority of ESR's Work-Life-Balance. Especially the subcategories “living” and “effective time management” suffered (Supplementary Figure 2). The data is clear that while secondments are invaluable in improving research skills and an interdisciplinary mindset, they are intense, taking a toll on ESRs by adding stress due to e.g., relocation to another country.

### 2.3 Scientific achievements

#### 2.3.1 Mission and structure of the TranSYS consortium

Although the global market offer promising job opportunities and sustainable career paths, there is a significant demand for skilled researchers who can bridge the interdisciplinary gap between life- and data/computational sciences in the industry. Consequently, the consortium at the core of TranSYS consisted of a combination of expertise in various fields like Systems Medicine, Functional genomics, Bioinformatics, Biostatistics, Integrative Biology, artificial intelligence (A.I.), Software Development, Blockchain, Ethics, and pharmacogenomics, completed by industry partners at the forefront of innovative future healthcare.

**Figure 4:**
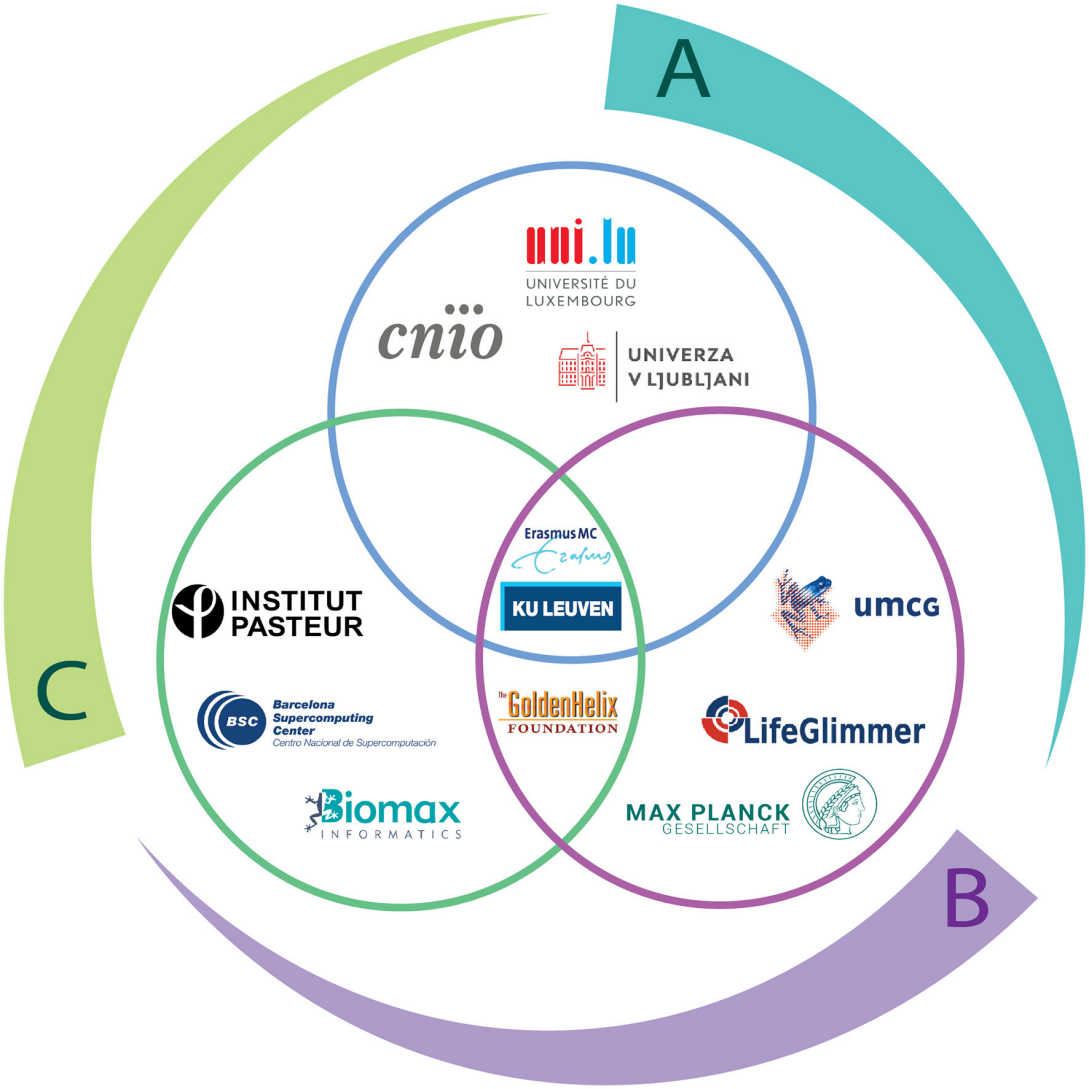
The structure of TranSYS. The outer circle represents the three key TranSYS objectives: (A) Narrowing the gap between preclinical performance and treatment benefit (B) Developing integrative strategies and corresponding integrated workflows (C) Improving understanding of patient heterogeneity and developing economically viable patient stratification strategies. The inner Venn Diagram illustrates the three work packages of TranSYS and the beneficiaries contributing to each of them: WP1 (blue) *Preclinical Science and Molecular Medicine*, WP2 (green) *Systems Analytics*, and WP3 (purple) *Targeted Therapeutics*.

An eagle-eye view of the structure of TranSYS can be found in Figure 4. Projects were outlined and designed by beneficiaries according to their research areas and capabilities, in order to achieve the following three main goals through interdisciplinary collaborations:

*A) Narrowing the gap between preclinical performance and treatment benefit* through the integration of preclinical wet-lab observations with *in-silico* modeling. The aim was to promote the development of more sophisticated disease progression models and significantly enhance discovery and validation of biomarkers. By bridging the gap between experimental and computational approaches, TranSYS ESRs could unlock a deeper understanding of diseases, leading to more effective diagnostic tools and targeted therapeutic interventions. Noteworthy among these efforts are the contributions of Najjary et al. (ESR3), who elucidated the omics on immune responses and immune system associated with the development of cancers (9). Additionally, Walakira et al. (ESR5), who applied genome-scale metabolic modeling to integrate transcriptomics data in a human reference model and extract personalized, context specific models for patients with HCC (10, 11). Wilson et al. (ESR6) dissected the cellular heterogeneity of Parkinson's disease related iPS cells during aging by multiomics to identify disease modifiers (manuscript in preparation). Furthermore, Berca et al. (ESR7) generated a full-characterized (at the histology, transcriptomic, genetic, and epigenetic level) biobank of PDO from bladder tumor samples and use them to study the underlying mechanisms driving resistance to Erdafitinib (manuscript in preparation).

*B) Developing integrative strategies and corresponding integrated workflows* by taking advantage of top-tier, state-of-the-art data resources across seven disease areas: inflammatory and autoimmune diseases, cancer, non-alcoholic fatty liver disease, neurodegenerative diseases, psychiatric disease, cardiovascular disease, and rare diseases. Cutting-edge data analysis approaches and innovative tools were employed to analyze intricate multi-level datasets, to gain unprecedented insights into these complex medical conditions, driving forward the understanding and treatment of these diseases. The pursuit of our research objectives has led to the development of ground breaking data analysis methodologies and innovative tools. For instance, Melograna et al. (ESR1) explored the potential of interaction, specifically of individual-specific networks or epistasis, to provide personalized insights on an individual's health or disease (12, 13). Li et al. (ESR2) developed a novel multi-view clustering pipeline based on networks and extraneous information (14). Andreoli et al. (ESR4) conducted a systematic review of reasons to identify the ethical and social implications related to the clinical use of polygenic risk scores. They identified a series of normative gaps to be urgently addressed before polygenic scores are implemented in the clinic (15). Yousefi et al. (ESR8) took advantage of network-based machine learning approaches to analyse clinical variation and capturing the dynamics of microbiome data (16–18). Mihajlovic et al. (ESR9) developed novel machine-learning algorithms based on non-negative matrix tri-factorization (NMTF) for integrating and mining time-series single cell transcriptomics data of Parkinson's disease cell line and a corresponding control with molecular networks and bulk omics data. They aimed to uncover novel disease-associated genes, emphasize mechanisms related to disease progression and propose treatment strategies based on drug-repurposing [(19), second manuscript in preparation]. The integration method was adapted and used to gain further insights into NRAS mutant melanoma cells adaptation to treatment (20). Lalli (ESR10) explored the significance of interoperability in model and data integration, spanning multi-omics and multi-modal datasets, and the transformation of raw data into reusable structures. Taheri et al. (ESR11) identified aging biomarkers using network-specific MRI analysis (manuscript in preparation).

*C) Improving understanding of patient heterogeneity and developing economically viable patient stratification strategies* via the employment of innovative methods and the creation of novel tools to effectively preprocess and analyze the above-mentioned datasets. Through the meticulous examination of population heterogeneity, variations in disease progression and treatment responses could be discerned. These findings served as a foundation to establish criteria for patient stratification in complex disease areas, enabling more targeted and personalized approaches to treatment. Leveraging advanced techniques, ESRs contribute to a deeper understanding of these challenging medical conditions and pave the way for more effective and tailored healthcare interventions. Korshevniuk et al. (ESR12) are dedicated to develop tools for integaring eQTL summary statistics, creating a pipeline for coexpression QTL mapping combining it in a framework for large-scale federated co-eQTL mapping (21). Hryhorzhevska (ESR13) investigated the impact of genetic variants and glucocorticoid receptor activation on gene expression and DNA methylation and the relationship of these variants with disease risk (22). Karamperis et al. (ESR14) advanced clinical pharmacogenomics in Europe (23, 24) but also deciphered the complex interaction of pharmacogenomic variants linked to adverse drug events across diverse populations, highlighting their substantial influence on commonly prescribed medications (25). Katz et al. (ESR15) focused on the development of artificial intelligence models to capture patient characteristics utilizing a variety of data, spanning from biochemical measurements to molecular data (26–29).

While the scientific ideas at the basis of TranSYS are formulated as these three main goals, they are executed in the form of three work packages, namely Preclinical Science and Molecular Medicine (WP1), Systems Analytics (WP2), and Targeted Therapeutics (WP3). For more detailed information on the strategies formulated within each work package can be found in the Supplementary Note 1.

#### 2.3.2 Case studies: how collaboration within TranSYS enabled scientific success

**Figure 5:**
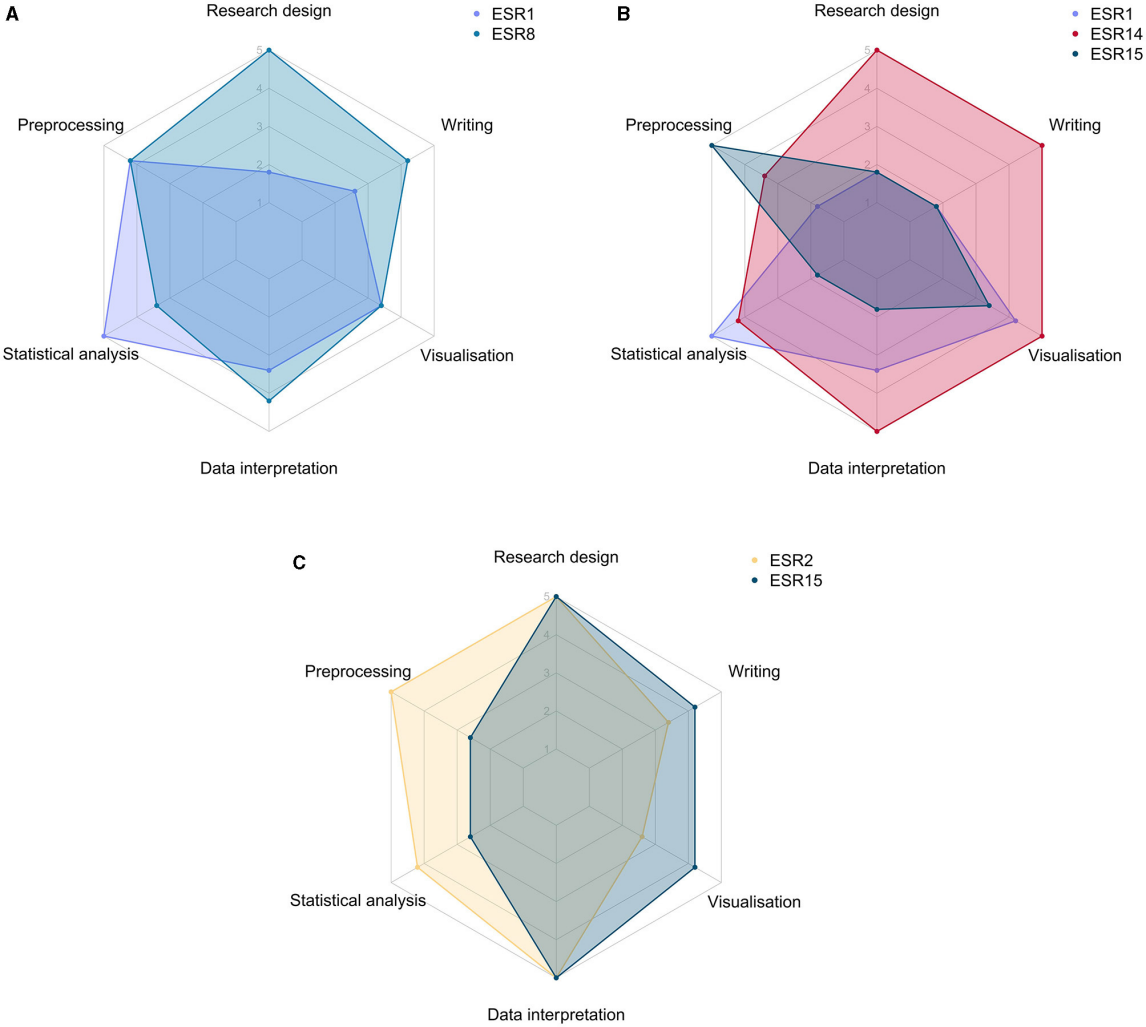
Graphical representation on how ESRs collaborated in successful TranSYS projects. **(A)** Capturing the dynamics of microbial interactions through individual-specific networks; **(B)** A worldwide spectrum of clinically relevant SNPs associated with drug toxicity: Genetic structure and risk characterization using population sequencing data to unravel geographical patterns and spatial trends in prescribed medications; **(C)** Deconfounding variational autoencoders for multi-omics data.

Collaboration, communication, and scientific exchange are values standing at the core of each ITN. TranSYS was no exception to this, and in the following paragraphs we attempt to give a selection of three diverse examples on how collaboration amongst ESRs facilitated projects that would have otherwise been deemed unfeasible; a graphical representation can be found in Figure 5. A more complete overview of the scientific output of the consortium can be found in Supplementary Table 1.

##### 2.3.2.1 Capturing the dynamics of microbial interactions through individual-specific networks

*Behnam Yousefi (ESR8), Federico Melograna (ESR1)* (16, 17)

In a perfect example of how the expertise of ESRs can be combined to tackle prior unfeasible projects, ESR1 and ESR8 joined forces to tackle the challenge of personalizing the analysis of temporal microbiome data (16). The analysis of individual-specific microbiome profiles over time or across conditions is an underrepresented topic in the field, due to the sheer complexity of the data. Combining the scientific vision and programming skills of ESR8 with the expertise of ESR1 in individual specific networks and statistics, Yousefi et al. were able to propose a out-of-the-box way of handling microbiome data, which they refer to as multiplex network differential analysis (MNDA). MNDA is able to resolve microbial dynamic patterns by combining representation learning and individual-specific microbial co-occurrence networks, ultimately uncovering taxon neighborhood dynamics. Supporting the movement of open science, MNDA is publicly available as an R package (17).

##### 2.3.2.2 A worldwide spectrum of clinically relevant SNPs associated with drug toxicity: genetic structure and risk characterization using population sequencing data to unravel geographical patterns and spatial trends in prescribed medications

*Kariofyllis Karamperis (ESR14), Sonja Katz (ESR15), Federico Melograna (ESR1); manuscript submitted*

The rapidly progressing field of personalized pharmacogenomics presents a significant potential to transform global approaches to patient drug treatments. Under the scientific guidance of a lead researcher (ESR14) and with collaborative efforts from two fellow researchers (ESR1 and ESR15), endeavors were made to identify, categorize, and analyze genetic variations associated with drug-induced toxicity. This initiative laid the foundation for the investigation of the prevalence of these risk-associated genetic alleles on a global scale, offering potential guidelines tailored to specific populations, which could be valuable for regulatory agencies. This collaborative endeavor serves as a compelling example of how the combined expertise of researchers with backgrounds in biology and computational sciences can culminate in a project of substantial significance for both national and international stakeholders. While the biological objectives were formulated by ESR14, the comprehensive analysis of genomic variants was made possible through the data mining proficiency of ESR15. Furthermore, the statistical and programming acumen contributed by ESR1 enhanced the project's communication by generating clear and information-rich visual representations of the findings.

##### 2.3.2.3 Deconfounding variational autoencoders for multi-omics data

*Zuqi Li (ESR2), Sonja Katz (ESR15)* (28)

The advent of high-throughput sequencing and the analysis of different types of “omics” data has shifted paradigms in biology and medicine. Today the field has developed toward multi-omics approaches, which follows the idea of using ever more data from different sources, aiming to be rewarded with a holistic picture of e.g., the disease under study. However, the integration of multiple data types, especially from different domains, is not trivial and biological meaningful analysis is hindered by another problem well known in epidemiology: confounding. Currently available data integration frameworks are able to either accommodate multiple data types (from different domains) or correct for biological confounders. ESR2 and ESR15 aim to address this shortcoming by merging these two, currently mutually exclusive, concepts. By combining their expertise in confounder adjustment and clustering (ESR2) and knowledge on developing deep learning models (ESR15), the two ESRs were able to implement a variety of deconfounding deep learning frameworks. This collaboration shows the potential held in matching ESRs strong computational backgrounds and complementary research interests; advanced *in-silico* projects yielding tools that may benefit the scientific community beyond the boundaries of TranSYS can be realized.

## 3 Discussion

The continuous progression of basic biomedical research, drug discovery and clinical applications, is allowing the implementation of PM in modern healthcare. Today, PM is widely recognized as an integral component of our future healthcare landscape. This realization has brought about a relevant shift in strategy, transforming PM from a scientifically-driven “bottom-up” development that forged its own path, to a “top-down” approach where decision-makers and healthcare systems actively facilitate PM-based approaches. The “bottom-up” approach often relies on regional networks, as exemplified by Sweden and Germany (30). On the other hand, the “top-down” approach involves national genome initiatives funded by governments, as seen in countries like England, France, and Denmark (31). This complementary combination of approaches has fostered the integration of PM into mainstream healthcare, ensuring its widespread implementation and impact. A European Partnership for Personalized Medicine was launched in October 2023 to boost research in precision medicine across the European Research Area. This builds on the activities of the International Consortium for Personalized Medicine (ICPerMed) action plan. This partnership will implement the Strategic Research and Innovation Agenda for Personalized Medicine, developed in collaboration with the European Commission. Achieving this ambition and taking a “Systems of Health” approach will require all key players to interact to deliver the societal benefits of PM to patients, citizens and society. At its core this PM strategy needs highly skilled researchers trained in interdisciplinary cross-sector environments, with collaborative cultures and creative mindsets, as pioneered by the TranSYS network and first cohort of researchers (32).

In the past two decades, remarkable strides have been made in the advancement and implementation of PM. During this period, we have observed the rapid emergence of high-throughput genomic technologies and big data analytics, initially employed in research to uncover the intricacies of disease mechanisms, and later adopted in clinical settings as potent diagnostic tools (1). Ongoing directions of biomedical research involve analyzing phenomena that occur across multiple scales, from molecular interactions to cellular processes and whole organism behavior. This has also underpinned a deepening understanding of disease and patient heterogeneity and tools to stratify patient populations and cluster diseases with similar aetiologies. Concurrently, novel targeted therapies have been developed, focusing on specific pathomechanisms, particularly in the context of cancer and rare diseases (33). This progress has paved the way for enhanced diagnostics and personalized treatment, representing a significant change from the traditional “one-size-fits-all” approach to precision healthcare (34). To tackle these intricate systems effectively, a holistic approach is required, where researchers from diverse disciplines collaborate and pool their expertise.

MSCA Training Networks have established themselves as a valuable part of the Horizon Europe Framework that aims at developing a skilled workforce capable of driving innovation especially in interdisciplinary fields, including PM. The TranSYS ITN was established under the previous Horizon 2020 programme offers young researchers the opportunity to gain multilayered training, engage in collaborative research projects, and develop novel techniques and tools revolving around personalized medicine.

### 3.1 ESRs showed clear improvement in scientific, professional, and social skills relatable to actions within the ITN

To give a more in-depth view on an ITNs most important asset—the researchers—we evaluated how the opportunities within TranSYS impacted the personal development of its 15 ESR. Across the 4 year ITN duration each ESR delivered a core 36 month individual research project and honed their skills in a range of different categories, showing a positive learning trend in 5 out of 6 assessed categories.

Unsurprisingly, amongst the expertise that can be clearly linked to ITN activities, research and communication skills showed the highest degree of improvement. The interdisciplinary nature of an ITN results in the need of clear communication with peers from different scientific fields and sectors. The networks also provide a platform that lends itself to constantly exposing researchers to new knowledge which drives the improvement of research-related skills. Our findings align with a recent evaluation study of the European framework programmes for research and innovation for excellent science, which found that around 80% of ITN fellows consider training options to be good or very good (35). However, we found that not all skill improvements could be attributed to direct ITN training activities. Some, such as management and leadership skills or professionalism, including skills such as networking or maintaining positive work relationships, rather evolve naturally in the course of doing a PhD project that is at the core of the ITN. Very notably, the category “Work-Life-Balance” entailing ratings on maintaining a healthy time management, keeping personal motivation or physical and emotional wellbeing, showed no improvement and could even be negatively associated with the mobility required within an ITN. The implications that can be learned from this finding are further discussed in the Open Challenges within an ITN section of the Discussion.

Although the statistical power of our analysis is limited due to the small number of ESRs, we found that the key training points within an ITN–scientific excellence and advanced communication skill in an increasingly interconnected world—are well reflected in the skill improvement for ESRs.

### 3.2 TranSYS stood out in terms of the large quantity and quality of research outputs

The recent evaluation study of the European framework programmes report a remarkably high quantity and quality of research originating from MSCA actions, and measure an expansion of the professional network due to collaborations within consortia (35). The scientific achievements of TranSYS reflect that general finding well—at the time of writing this manuscript, a total of 17 different datasets or computational tools have been generated. As of today, the combined number of publications anticipated at the conclusion of the PhD for all ESRs is expected to surpass 50, with the majority of these manuscripts currently in various stages of preparation, submission, or revision. The high number of collaborations outside of the planned secondments paint a picture of extensive collaboration and networking within TranSYS, often initiated by the ESRs. Our selection of *collaboration success stories* clearly illustrate what collaboration within TranSYS looked like, how it was facilitated through the skills of individuals, and which scientific outcomes were delivered. We believe these showcases underline the spirit and opportunities within ITNs, as none of these (and other) projects would have been feasible for individual students, but were facilitated—or even positively reinforced—within TranSYS.

### 3.3 The impact of TranSYS outside academia

EU Framework Programmes, including Horizon 2020, promote several concepts to define societal impact, amongst them the (i) better contribution of research to tackling societal challenges and the (ii) better societal acceptance of science and innovative solutions (4, 35). We argue that TranSYS ESRs have significantly contributed to both points, with their focus on tools advancing personalized medicine, with the intention of improved patient wellbeing. TranSYS featured ESRs solely dedicated to aspects including responsible data storing and model sharing through federated learning and blockchain technology (ESR10) or ethical questions and stakeholders' perspectives on the clinical use of genomic data in clinics (ESR4). With a mindsets toward open and data-driven research and development, all manuscripts by ESRs were published open access, and the underlying data or code is available publicly or upon request. In terms of societal acceptance, ESRs actively participated in a number of outreach and science communication activities. However, ITN outreach activities largely remained on a local and individual level due to the costs and need for having an established name associated with some activities, e.g., international science fairs. To broaden the societal impact of MSCA actions and help bring ITNs out of academia and into society, we feel the need for dedicating parts of the budget toward funding a joint ITN outreach activity. This could be of larger-scale and marketed more effectively than the individual small outreach activities currently organized.

### 3.4 Open challenges within an ITN

In the spirit of adding to the continuous development of the MSCA Doctoral Training Networks, we provide a critical view on current bottlenecks in ITNs, as perceived by ESRs, and give suggestions on how these can be best addressed to further improve the experience of future ESRs.

#### 3.4.1 The act of balancing interests

By its nature, ITNs are consortia with a variety of stakeholders–universities, academic partners, industry beneficiaries and others—each with unique interests that sometimes can not be easily aligned. Despite the unique opportunities offered by this mix of stakeholders, it can result in a demanding and straining program for ESRs struggling to balance university requirements for obtaining a PhD degree, the scientific goals of their supervising PIs, and the competitiveness of industry partners. Secondments with industry partners not hosting ESRs were found to be challenging; a lack of awareness of the project and skills of visiting ESRs, no clear objectives for secondment projects, or the clash of intellectual property interests preventing company knowledge sharing with ESRs who could have contributed positively to further developments in the company. This balancing act is not unique to ITNs within the field of personalized medicine—another biotechnology-focused ITN called YEASTCELL found that the lack of a common ground in industry academia cooperation may jeopardize the success of entire ESR projects (3, 36). To avoid conflicts of interest, they must be identified early in the project planning phase and all stakeholders have to actively communicate their interests to achieve a set of focused and shared ITN objectives. For example, to facilitate the academic requirements of ESRs, doctoral schools enrolling ESRs should acknowledge ITN training with ECTS. Also, industry partners should be given the chance for direct input into the design of the research programme, this secures stronger industry engagement, and a stronger partnership with less potential for conflicts of interest.

#### 3.4.2 Mobility: a challenging gift

A key point making ITNs such exceptional programmes for young researchers are their mobility opportunities. During secondments, ESRs have the possibility to visit other partners from within the network for extended periods, of up to 6 months, to receive training and collaborate in person—an endeavor which is fully funded by the beneficiary institution that employs the ESR. While mobility is the most prestigious part of ITNs and our study can link successful secondments to an improvement in scientific and professional expertise, we also find that they have a negative impact on the quality of life of ESRs (Figure 3B). Although aspiring researchers are aware of the mobility requirements within ITNs prior to starting the position, it is easily underestimated the scale of the disruption to life. TranSYS included two secondments within 3 years that each lasted several weeks and involved relocations. A majority of the difficulties accompanying secondments arise from the challenges associated with relocation to another country, for example extensions of visa allowances or finding short-term housing, or difficulties in continuing ongoing projects next to the new responsibilities within the secondment, especially in the case of wet-lab experiments. We argue that there is a clear need for counteracting the negative trends seen in the “Work-Life-Balance” category with (i) more workshops focused on acquiring skills to improve the quality of life of ESRs and (ii) a stronger sense of responsibility from guest institutions for visiting ESRs. With the elevated rates of depression, burnout, and anxiety in PhD students being well documented (37) useful training included in an ITN may revolve around topics such as effective project management, mindful productivity, stress identification and management, or how to negotiate difficult topics with supervisors. To alleviate some of the stress associated with a secondment, a stronger sense of responsibility or even the formal commitment from hosting institutions to assist ESR is needed in the period leading up to their visit. This is already built into Doctoral Networks planning but based on the experience of ESRs a more proactive approach needs to be encouraged from the project kick-off. A local representative responsible for offering translating skills, communication with officials, or aiding in identifying adequate housing may prove invaluable for visiting ESRs.

#### 3.4.3 It takes a village–of not just PhD students

The personal perception of TranSYS being very successful in terms of collaboration, scientific output, and shared knowledge aligns well with the numbers identified in the course of this study. However, we argue that the degree of connectivity within TranSYS could have been promoted even more; while ESRs were highly connected through secondments, summer schools, or daily communication channels, the willingness to collaborate lacked amongst some PIs. The COVID-19 pandemic enforced restriction on traveling and in-person events, and while summer schools were mandatory for ESRs, the rules for PIs were less strict, often resulting in few of them joining events. This led to a number of initial project ideas not getting pursued, due to the lack of engagement by supervisors, or changes in the supervisory team leading to conflicts between initial project directions and changing supervisor interests. Collaborations also rarely extended beyond ESRs or PIs to the lab members of beneficiaries; this could be compounded by the individual nature of the PhD projects.

We can conclude that while a demanding programme, the opportunities provided within ITNs are well worth the effort and commitment required to succeed in the dynamic modern research landscape. However, we argue that ITNs are not without faults. Giving attention to some of the points outlined in this section can lead to the design and implementation of better Doctoral Networks in the future. The weaknesses identified can be mitigated without compromising the strengths of MSCA doctoral training programmes, ultimately making ITNs an even more attractive choice for aspiring future leaders.

### 3.5 Future prospects

Despite the majority of projects coming to a conclusion in 2023, TranSYS is far from ending. Currently most ESRs are in the process of working toward their PhD degrees and looking toward starting new positions in academia and industry all over Europe. However, the multidisciplinary network they built throughout the period of the ITN will not disappear—especially for ESRs seeking to stay in research. The connections and working relationships are certain to contribute toward a successful career start in academia. In the course of the last official consortium meeting, ESRs agreed to the organization of informal yearly reunions to maintain contact in a scientific, but also personal sense. We hope that this way the connections we built through TranSYS will not only stay strong, but even thrive.

## 4 Conclusion

Precision Medicine has undeniably become a cornerstone of our forthcoming healthcare landscape. In this context, MSCA training actions have firmly established themselves within the Horizon Europe Framework, as the driving force of the new generation of highly qualified scientists. Notably, the TranSYS ITN has showcased substantial enhancements in the expertise of its ESRs, spanning research insight and communication proficiencies. The inherent interdisciplinary nature of an ITN necessitates effective communication among peers from diverse scientific backgrounds. This environment also exposes ESRs to a continuous influx of novel knowledge, thereby fostering the refinement of research-related skills. Moreover, the ITN has yielded substantial collaborative opportunities that extend beyond planned secondments. It is crucial to underscore the social impact achieved through outreach and training initiatives, as these actions effectively bridge the gap between ITNs and society at large. Even though some challenges still remain to be overcome, the excellent outcomes derived from this ITN experience across several dimensions and reflected in this work, are progressively laying the foundation for robust networks capable of conducting exceptional basic and translational research for the future.

## 5 Materials and methods

### 5.1 Collection of self-reported evaluation metrics

To assess the development of ESRs during the course of TranSYS, a questionnaire was prepared covering various areas, including background and education, technical skills and competences, academic mobility, collaborations, and scientific output. We additionally utilized the self-assessment survey part of each ESR's Career Development Plan (CDP)—a mandatory tool within all ITNs to track ESR development—to assess the improvement of ESRs in a range of technical and soft skills. This self-assessment survey adapted from the University of Florida allows the evaluation of current strengths and weaknesses. Therefore, we asked ESRs to report their CDP assessment from two different time points: the first CDP filled upon starting the project, and the last CDP filled upon project end, ultimately covering a period of 3 years (with individual starting points). The self-assessment covered the following major categories, each made up by a number of minor categories and rated with scores from 1 (no knowledge) to 5 (expert) (Table 2). In an attempt to relate the impact of TranSYS activities (summer schools, bootcamps) on each minor skill category, we asked ESRs to rate the perceived impact of each activity on their development. The ordinal scale used for this purpose ranged from 1 (negative impact) to 10 (highly beneficial).

**Table 2:** Overview of major and minor skill categories assessed.

**Research skills**	**Work-life-balance**	**Professionalism**	**Management and leadership**	**Communication**	**Career advancement**
Broad-based knowledge of field	Maintaining openness and curiosity	Identifying and seeking advice	Providing instruction and guidance	Writing for experts in my field	Building transferable skills
Critical reading of literature in the field	Living (physical, emotional, financial)	Upholding commitments and deadlines	Providing constructive feedback	Writing for a lay audience	Identifying career options
Experimental and research design	Effective healthy time management	Maintaining positive relationships	Dealing with conflict	Grant writing skills	Preparing application or valorization materials
Careful record keeping practices	Maintaining personal motivation	Contributing to a team in the office or lab	Serving as a role model	Speaking clearly and effectively	Asking the right questions
Understanding data ownership	Fostering diversity of academic perspectives	Contributing outside the team	Delegating responsibilities in research setting	Using the right body language	Negotiating skills
Demonstrating responsible conduct in human or animal research and publications		Building and maintaining scientific network	Leading and motivating others	Editing own work	Participating in professional service
Identifying research misconduct				Carrying out peer review	Adopting long-term approach to career

### 5.2 Statistical analysis

For statistical analysis of data collected using the self-assessment survey, we aggregated the minor categories to obtain one skill rating per major category for each ESR. *P*-values for the comparison of 1^*st*^ and 3^*rd*^ year ratings were subsequently derived using a Wilcoxon signed-rank test and multiple testing corrected using Bonferroni correction. To investigate whether TranSYS events show an effect on the skill improvement of ESRs, a linear regression for each category was conducted. All analyses were carried out using R (version 4.3.1) and python (version 3.8.5).

## Data availability statement

The original contributions presented in the study are included in the article/Supplementary material, further inquiries can be directed to the corresponding author.

## Ethics statement

All participants in this study, who were also the co-authors, were fully informed about the research's purpose and procedures, ensuring transparency in the research process. Participants voluntarily signed a consent statement, explicitly approving the use of their questionnaire responses for research purposes. This procedure is supported by the TranSYS Ethics Committee.

## Author contributions

LA: Conceptualization, Investigation, Methodology, Project administration, Writing – original draft, Writing – review & editing. CB: Conceptualization, Investigation, Methodology, Project administration, Writing – original draft, Writing – review & editing. SK: Conceptualization, Formal analysis, Investigation, Methodology, Project administration, Visualization, Writing – original draft, Writing – review & editing. MK: Conceptualization, Formal analysis, Investigation, Methodology, Project administration, Visualization, Writing – original draft, Writing – review & editing. RH: Writing – review & editing. KV: Funding acquisition, Supervision, Writing – review & editing. TC: Supervision, Writing – review & editing.

## Group members in the TranSYS Consortium

Kristel Van Steen, Laboratory for Systems Genetics, GIGA-R Medical Genomics, University of Liège, Liège, Belgium; BIO3 - Laboratory for Systems Medicine, Department of Human Genetic, KU Leuven, Leuven, Belgium; Kris Dierickx, Department of Public Health and Primary Care, Centre for Biomedical Ethics and Law, KU Leuven, Leuven, Belgium; Lara Andreoli, Department of Public Health and Primary Care, Centre for Biomedical Ethics and Law, KU Leuven, Leuven, Belgium; Federico Melograna, BIO3 - Laboratory for Systems Medicine, Department of Human Genetic, KU Leuven, Leuven, Belgium; Zuqi Li, BIO3 - Laboratory for Systems Medicine, Department of Human Genetic, KU Leuven, Leuven, Belgium; Giada Lalli, BIO3 - Laboratory for Systems Medicine, Department of Human Genetic, KU Leuven, Leuven, Belgium; Johan M. Kros, Department of Pathology, The Tumor Immune-Pathology Laboratory, Erasmus University Medical Center, Rotterdam, Netherlands; Shiva Najjary, Department of Pathology, The Tumor Immune-Pathology Laboratory, Erasmus University Medical Center, Rotterdam, Netherlands; Damjana Rozman, Centre for Functional Genomics and Bio-Chips, Institute of Biochemistry and Molecular Genetics, Faculty of Medicinem University of Ljubljana, Ljubljana, Slovenia; Andrew Walakira, Centre for Functional Genomics and Bio-Chips, Institute of Biochemistry and Molecular Genetics, Faculty of Medicine, University of Ljubljana, Ljubljana, Slovenia; Alexander Skupin, Luxembourg Centre for Systems Biomedicine (LCSB), Université du Luxembourg; Elle Wilson, Luxembourg Centre for Systems Biomedicine (LCSB), Université du Luxembourg; Francisco X. Real, Epithelial Carcinogenesis Group, Spanish National Cancer Research Centre, Madrid, Spain; CIBERONC, Madrid, Spain; Department of Medicine and Life Sciences, Universitat Pompeu Fabra, Barcelona, Spain; Catalina Berca, Epithelial Carcinogenesis Group, Spanish National Cancer Research Centre, Madrid, Spain; Benno Schwikowski, Computational Systems Biomedicine Lab, Department of Computational Biology, Institut Pasteur, Paris, France; Behnam Yousefi, Computational Systems Biomedicine Lab, Department of Computational Biology, Institut Pasteur, Paris, France; Nataša Pržulj, Barcelona Supercomputing Center (BSC), Barcelona, Spain; Department of Computer Science, University College London, London WC1E 6BT, United Kingdom; ICREA, Pg. Lluis Companys, Barcelona, Spain; Katarina Mihajlovic, Barcelona Supercomputing Center (BSC), Barcelona, Spain; Department of Computer Science, Universitat Politècnica de Catalunya BarcelonaTech (UPC), Campus Nord, Building Ω (Omega), C. Jordi Girona, Barcelona, Spain; Elisabeth B. Binder, Department of Psychiatry and Behavioral Sciences, Emory University School of Medicine, Atlanta, GA, United States; Department Genes and Environment, Max Planck Institute of Psychiatry, Munich, Germany; Nahid Taheri, Department Genes and Environment, Max Planck Institute of Psychiatry, Munich, Germany; Anastasiia Hryhorzhevska, Department Genes and Environment, Max Planck Institute of Psychiatry, Munich, Germany; Lude Franke, Department of Genetics, University of Groningen, University Medical Center Groningen, Groningen, Netherlands; Oncode Institute, Utrecht, Netherlands; Maryna Korshevniuk, Department of Genetics, University of Groningen, University Medical Center Groningen, Groningen, Netherlands; George P. Patrinos, University of Patras School of Health Sciences, Department of Pharmacy, Division of Pharmacology and Biosciences, Laboratory of Pharmacogenomics and Individualised Therapy, Patras, Greece; Department of Genetics and Genomics, College of Medicine and Health Sciences, United Arab Emirates University, Al-Ain, Abu Dhabi, United Arab Emirates; Zayed Centre for Health Sciences, College of Medicine and Health Sciences, United Arab Emirates University, Al-Ain, Abu Dhabi, United Arab Emirates; Erasmus University Medical Centre, Faculty of Medicine and Health Sciences, Department of Pathology—Clinical Bioinformatics Unit, Rotterdam, Netherlands; Kariofyllis Karamperis, Laboratory of Pharmacogenomics and Individualized Therapy, Department of Pharmacy, School of Health Sciences, University of Patras, Patras, Greece; The Golden Helix Foundation, London, United Kingdom; Laboratory of Algorithms for Population Genomics, Department of Genetics, Institut de Biologia Evolutiva, IBE, (CSIC-Universitat Pompeu Fabra), Barcelona, Spain; Oscar Lao, Laboratory of Algorithms for Population Genomics, Department of Genetics, Institut de Biologia Evolutiva, IBE, (CSIC-Universitat Pompeu Fabra), Barcelona, Spain; Vitor A. P. Martins dos Santos LifeGlimmer GmbH, Berlin, Germany; Laboratory of Bioprocess Engineering, Wageningen University & Research, Wageningen, Netherlands; Sonja Katz, LifeGlimmer GmbH, Berlin, Germany; Laboratory of Systems and Synthetic Biology, Wageningen University & Research, Wageningen, Netherlands; Department of Radiology and Nuclear Medicine, Erasmus MC, Rotterdam, Netherlands; Christina Olsen, Ceratium BV, Amsterdam, Netherlands; Ritchie Head, Ceratium BV, Amsterdam, Netherlands.
