# Supplementary material for: Bridging the gap in precision medicine: TranSYS training programme for next-generation scientists
Source: Front Med (Lausanne). 2024 May 24;11:1348148. doi: 10.3389/fmed.2024.1348148 (PMC11160483; doi:10.3389/fmed.2024.1348148)

Supplementary Material

Bridging the Gap in Precision Medicine: TranSYS Training Programme for Next-Generation Scientists

Lara Andreoli [1,†], Catalina Berca [2,†], Sonja Katz [3,4,5,†], Maryna Korshevniuk [6,†], Ritchie M. Head [7], Kristel van Steen [8,9,*], and the TranSYS consortium

[1]Department of Public Health and Primary Care, Centre for Biomedical Ethics and Law, KU Leuven, Leuven, Belgium
[2]Epithelial Carcinogenesis Group, Molecular Oncology Programme, Spanish National Cancer Research Centre (CNIO), Madrid, Spain
[3]Laboratory of Systems and Synthetic Biology, Wageningen University and Research, Wageningen, The Netherlands
[4]Department of Radiology and Nuclear Medicine, Erasmus MC, Rotterdam, The Netherlands
[5]LifeGlimmer GmbH, Berlin, Germany
[6]Genetics Department, University Medical Center Groningen, Groningen, The Netherlands
[7]Ceratium BV, Amsterdam, The Netherlands
[8]Laboratory for Systems Genetics, GIGA-R Medical Genomics, University of Liege, Liege, Belgium
[9]Laboratory for Systems Medicine, Department of Human Genetics, KU Leuven,Leuven, Belgium
[†]These authors contributed equally to this work and share first authorship

Supplementary Note 1: **Work package description**

WP1 - Preclinical Science and Molecular Medicine
WP1 focuses on understanding disease mechanisms through experimental and biological sciences, including cell biology, physiology, chemistry, microbiology, and medical genomics. The goal is to identify biomarkers with predictive, diagnostic, or prognostic abilities by leveraging data generation technologies and merging lab science with computational research.

WP2 - Systems Analytics
WP2 concentrates on diverse modeling viewpoints, including mathematical, dynamical, geometric, statistical, and AI-based approaches. The focus is on integrating data from various sources in a systematic manner to effectively manage complex diseases, like neurodegenerative disorders.

WP3 - Targeted Therapeutics
WP3 bridges scientific discoveries and clinical applications in areas such as pharmacology, pharmacogenomics, functional genomics, and systems genomics. The aim is to translate knowledge into practical applications, identifying patient heterogeneity's implications for disease prevention, pre-emption, and treatment. Research tools are developed to aid patient selection for optimized disease management and treatment benefits.

Supplementary Table 1: **Overview on TranSYS publications** (as of November 2023)

ESR	Title	Year	DOI
1	Gregorich M, Melograna F, Sunqvist M, Michiels S, Van Steen K, Heinze G. **Individual-specific networks for prediction modelling–a scoping review of methods.** BMC Medical Research Methodology. 2022 Mar 6;22(1):62.	2022	10.1186/s12874-022-01544-6
1	Yousefi B, Melograna F, Galazzo G, van Best N, Mommers M, Penders J, Schwikowski B, Van Steen K. **Capturing the dynamics of microbial interactions through individual-specific networks**. Frontiers in Microbiology. 2023 May 15;14:1170391.	2023	10.3389/fmicb.2023.1170391
1	Melograna F, Li Z, Galazzo G, van Best N, Mommers M, Penders J, Stella F, Van Steen K. **Edge and modular significance assessment in individual-specific networks**. Scientific Reports. 2023 May 15;13(1):7868.	2023	10.1038/s41598-023-34759-8
2	Li Z, Melograna F, Hoskens H, Duroux D, Marazita ML, Walsh S, Weinberg SM, Shriver MD, Müller-Myhsok B, Claes P, Van Steen K. **netMUG: a novel network-guided multi-view clustering workflow for dissecting genetic and facial heterogeneity**. Frontiers in genetics. 2023 Dec 6;14:1286800.	2023	10.3389/fgene.2023.1286800
2	Li Z, Katz S, Saccenti E, Fardo DW, Claes P, Martins dos Santos VA, Van Steen K, Roshchupkin GV. **Novel multi-omics deconfounding variational autoencoders can obtain meaningful disease subtyping.** bioRxiv. 2024 Feb 8:2024-02.	2023	doi.org/10.1101/2024.02.05.578873
3	Najjary S, Mustafa DA, Kros JM. **Non-small cell lung cancer brain metastasis: the link between molecular mechanisms and novel therapeutic approaches.** Cancer Metastasis-Molecular Mechanism and Clinical Therapy. 2022 Oct 4.	2022	10.5772/intechopen.106385
3	Najjary S, Kros JM, Stricker BH, Ruiter R, Shuai Y, Kraaij R, Van Steen K, van der Spek P, Van Eijck CH, Ikram MA, Ahmad S. **Association of blood-cell-based inflammatory markers with gut microbiota and cancer incidence in the Rotterdam Study.**	2023	10.21203/rs.3.rs-3110898/v1
4	Andreoli L, Peeters H, Van Steen K, Dierickx K. **Taking the risk. A systematic review of ethical reasons and moral arguments in the clinical use of polygenic risk scores.**	2023	10.1002/ajmg.a.63584

	American Journal of Medical Genetics Part A. 2024 Mar 7:e63584.		
5	Walakira A, Rozman D, Režen T, Mraz M, Moškon M. **Guided extraction of genome-scale metabolic models for the integration and analysis of omics data.** Computational and Structural Biotechnology Journal. 2021 Jan 1;19:3521-30.	2021	10.1016/J.CSBJ.2021.06.009
5	Walakira A, Ocira J, Duroux D, Fouladi R, Moškon M, Rozman D, Van Steen K. **Detecting gene–gene interactions from GWAS using diffusion kernel principal components**. Bmc Bioinformatics. 2022 Dec;23(1):1-8.	2022	10.1186/S12859-022-04580-7
5	Walakira A, Skubic C, Nadižar N, Rozman D, Režen T, Mraz M, Moškon M. **Integrative computational modeling to unravel novel potential biomarkers in hepatocellular carcinoma**. Computers in Biology and Medicine. 2023 Jun 1;159:106957.	2023	10.1016/j.compbiomed.2023.106957
8	Firoozbakht F, Yousefi B, Schwikowski B. **An overview of machine learning methods for monotherapy drug response prediction**. Briefings in Bioinformatics. 2022 Jan;23(1):bbab408..	2022	10.1093/bib/bbab408
8	Yousefi B, Firoozbakht F, Melograna F, Schwikowski B, Van Steen K. **PLEX. I: a tool to discover features in multiplex networks that reflect clinical variation.** Frontiers in Genetics. 2023 Oct 19;14:1274637.	2023	10.3389/fgene.2023.1274637
8	Yousefi B, Melograna F, Galazzo G, van Best N, Mommers M, Penders J, Schwikowski B, Van Steen K. **Capturing the dynamics of microbial interactions through individual-specific networks.** Frontiers in Microbiology. 2023 May 15;14:1170391.	2023	10.3389/fmicb.2023.1170391
8	Yousefi, B., & Schwikowski, B. (2024)**. Consensus Clustering for Robust Bioinformatics Analysis**. *bioRxiv*, 2024-03.	2024	doi.org/10.1101/2024.03.21.586064
9	Gureghian V, Herbst H, Kozar I, Mihajlovic K, Malod-Dognin N, Ceddia G, Angeli C, Margue C, Randic T, Philippidou D, Nomigni MT. **A multi-omics integrative approach unravels novel genes and pathways associated with senescence escape after targeted therapy in NRAS mutant melanoma.** Cancer gene therapy. 2023 Jul 7:1-6.	2023	10.1038/s41417-023-00640-z
9	Mihajlović, K., Ceddia, G., Malod-Dognin, N., Novak, G., Kyriakis, D., Skupin, A., & Pržulj, N. (2023). **Multi-omics integration of scRNA-seq time series data predicts new intervention points for Parkinson's disease**. *bioRxiv*, 2023-12.	2023	doi.org/10.1101/2023.12.12.570554

10	Farah I, Lalli G, Baker D, Schumacher A. **A global omics data sharing and analytics marketplace: case study of a rapid data COVID-19 pandemic response platform.** medRxiv. 2020 Sep 29:2020-09.	2020	10.1101/2020.09.28.20203257
10	**ISN-tractor: a python library for the fast and scalable computation of biologically meaningful Individual-Specific Networks** Oxford Bioinformatics Journal Lalli, Giada; KU Leuven, Human Genetics Li, Zuqi; KU Leuven, Human Genetics Melograna, Federico; KU Leuven, Human Genetics Collier, James; VIB, Bioinformatics Core Moreau, Yves; KU Leuven Raimondi, Daniele; KU Leuven, ESAT-STADIUS Vansteen, Kristel; Universite de liege, Montefiore Institute/GIGA; KU Leuven, Human Genetics	2024	10.21203/rs.3.rs-3991527/v1
12	Li S, Schmid KT, de Vries DH, Korshevniuk M, Losert C, Oelen R, van Blokland IV, BIOS Consortium, sc-eQTLgen Consortium, Groot HE, Swertz MA, van der Harst P. **Identification of genetic variants that impact gene co-expression relationships using large-scale single-cell data**. Genome Biology. 2023 Apr 18;24(1):80.	2023	10.1186/s13059-023-02897-x
13	Knauer-Arloth J, Hryhorzhevska A, Binder EB. **Multi-omics analysis of the molecular response to glucocorticoids-insights into shared genetic risk from psychiatric to medical disorders.** medRxiv. 2023 Dec 5:2023-12.	2023	10.1101/2023.12.05.23299430
14	Stratopoulos A, Kolliopoulou A, Karamperis K, John A, Kydonopoulou K, Esftathiou G, Sgourou A, Kourakli A, Vlachaki E, Chalkia P, Theodoridou S. **Genomic variants in members of the Krüppel-like factor gene family are associated with disease severity and hydroxyurea treatment efficacy in β-hemoglobinopathies patients.** Pharmacogenomics. 2019 Jun;20(11):791-801..	2019	10.2217/pgs-2019-0063
14	Siamoglou S, Karamperis K, Mitropoulou C, Patrinos GP. **Costing methods as a means to measure the costs of pharmacogenomics testing. The Journal of Applied Laboratory Medicine**. 2020 Sep;5(5):1005-16.	2020	10.1093/jalm/jfaa113
14	Stathoulias A, Milioni A, Kritikou S, Karmakolia A, Goudoudaki S, Siamoglou S, Chassomeris C, Vassilakis S, Karamperis K, Velegraki A, Anastassopoulou C. **Toward high-throughput fungal electroculturomics and new omics methodologies in 21st-century microbiology and ecology**. OMICS: A Journal of Integrative Biology. 2020 Aug 1;24(8):493-504.	2020	10.1089/omi.2020.0012

14	Karamperis K, Koromina M, Papantoniou P, Skokou M, Kanellakis F, Mitropoulos K, Vozikis A, Müller DJ, Patrinos GP, Mitropoulou C. **Economic evaluation in psychiatric pharmacogenomics: a systematic review. The pharmacogenomics journal.** 2021 Aug;21(4):533-41.	2021	10.1038/s41397-021-00249-1
14	Karamperis K, Tsoumpeli MT, Kounelis F, Koromina M, Mitropoulou C, Moutinho C, Patrinos GP. **Genome-based therapeutic interventions for β-type hemoglobinopathies. Human Genomics**. 2021 Dec;15(1):1-8.	2021	10.1186/s40246-021-00329-0
14	Koufaki MI, Karamperis K, Vitsa P, Vasileiou K, Patrinos GP, Mitropoulou C. **Adoption of pharmacogenomic testing: a marketing perspective.** Frontiers in Pharmacology. 2021 Sep 17;12:724311.	2021	10.3389/fphar.2021.724311
14	Kambouris ME, Goudoudaki S, Kritikou S, Milioni A, Karamperis K, Giavasis I, Patrinos GP, Velegraki A, Manoussopoulos Y. **Beyond the Microbiome: Germ-ganism? An Integrative Idea for Microbial Existence, Organization, Growth, Pathogenicity, and Therapeutics**. OMICS: A Journal of Integrative Biology. 2022 Apr 1;26(4):204-17.	2022	10.1089/omi.2022.0015
15	Katz S, Suijker J, Hardt C, Madsen MB, Meij-de Vries A, Pijpe A, Skrede S, Hyldegaard O, Solligård E, Norrby-Teglund A, Saccenti E. **Decision support system and outcome prediction in a cohort of patients with necrotizing soft-tissue infections.** International Journal of Medical Informatics. 2022 Nov 1;167:104878.	2022	10.1016/j.ijmedinf.2022.104878
15	Katz S, Martins dos Santos VA, Saccenti E, Roshchupkin GV. **mEthAE: an Explainable AutoEncoder for methylation data.** bioRxiv. 2023:2023-07.	2023	10.1101/2023.07.18.549496
15	Kočar E, Katz S, Pušnik Ž, Bogovič P, Turel G, Skubic C, Režen T, Strle F, Dos Santos VA, Mraz M, Moškon M. **COVID-19 and cholesterol biosynthesis: Towards innovative decision support systems.** Iscience. 2023 Oct 20;26(10).	2023	10.1016/j.isci.2023.107799

Supplementary Figure 1: **Skill improvement for each minor category**

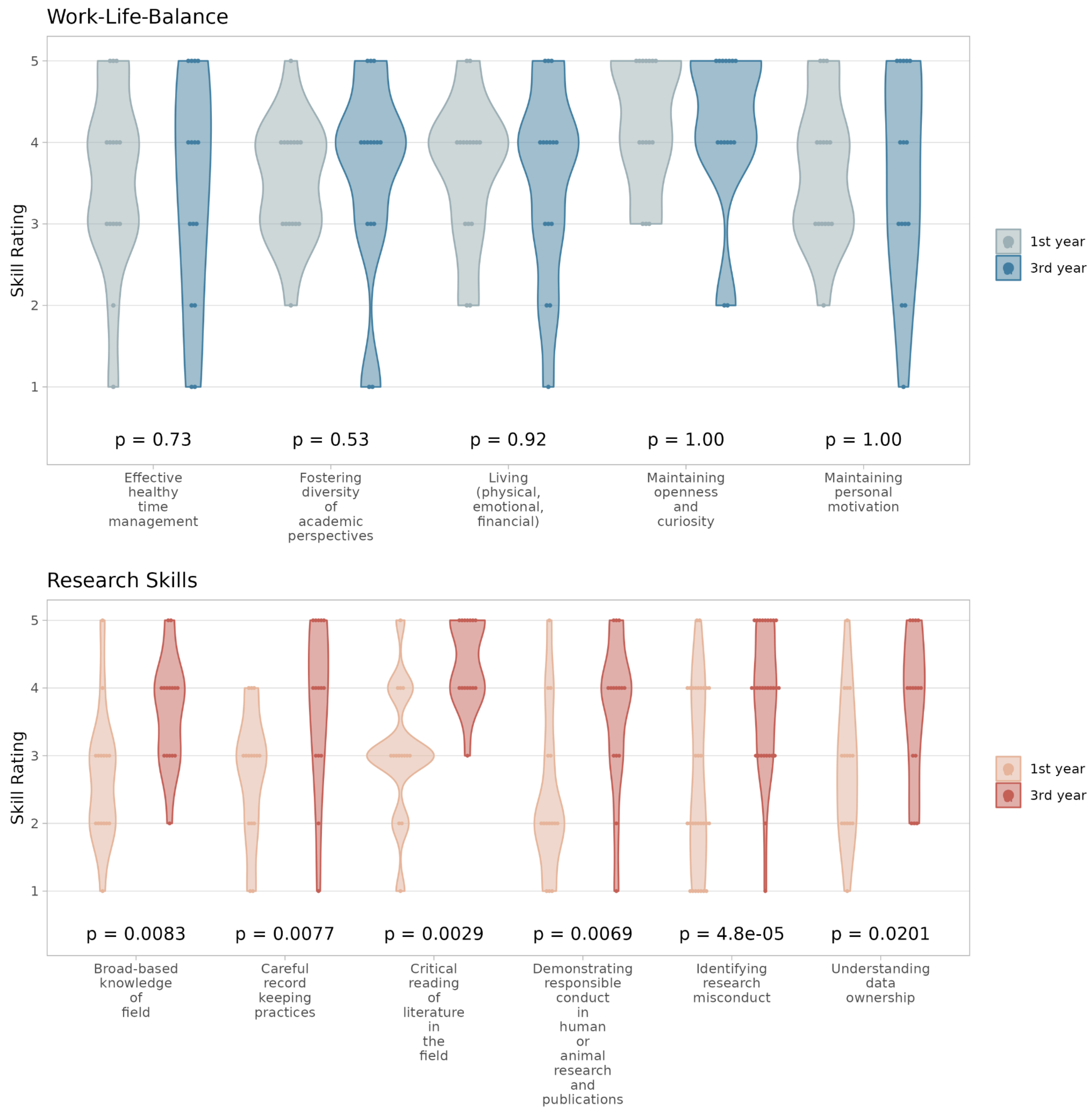

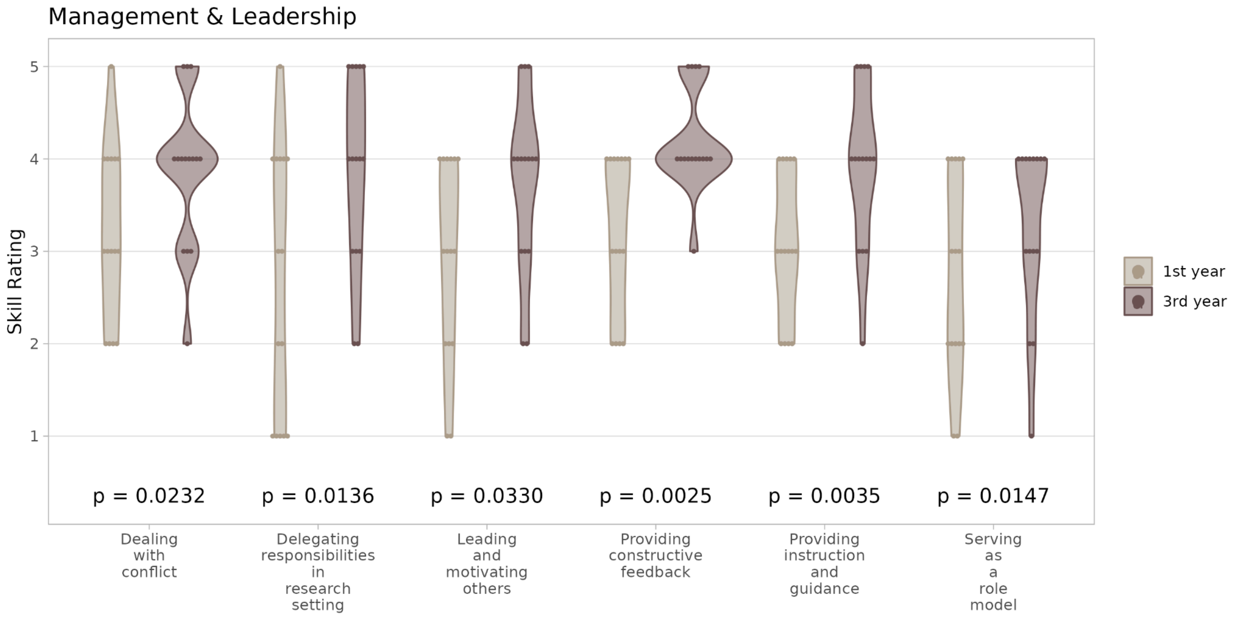
Management & Leadership
Skill Rating
5
4
3
2
1
p = 0.0232
p = 0.0136
p = 0.0330
p = 0.0025
p = 0.0035
p = 0.0147
Dealing with conflict
Delegating responsibilities in research setting
Leading and motivating others
Providing constructive feedback
Providing instruction and guidance
Serving as a role model
1st year
3rd year

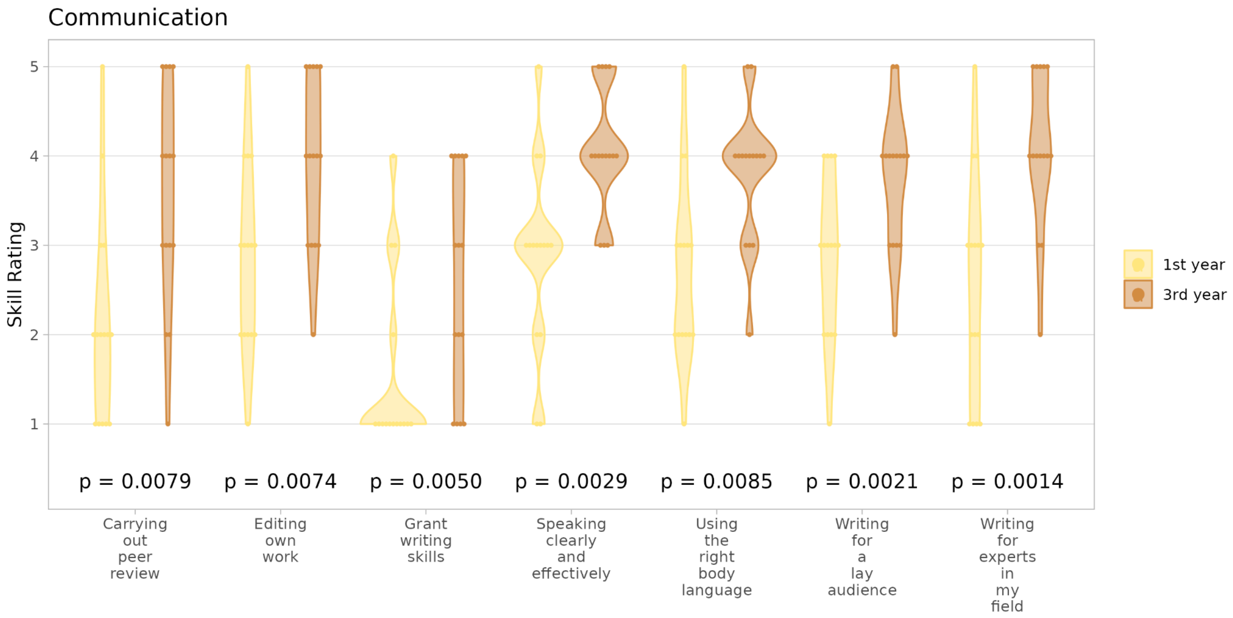
Communication
Skill Rating
5
4
3
2
1
p = 0.0079
p = 0.0074
p = 0.0050
p = 0.0029
p = 0.0085
p = 0.0021
p = 0.0014
Carrying out peer review
Editing own work
Grant writing skills
Speaking clearly and effectively
Using the right body language
Writing for a lay audience
Writing for experts in my field
1st year
3rd year

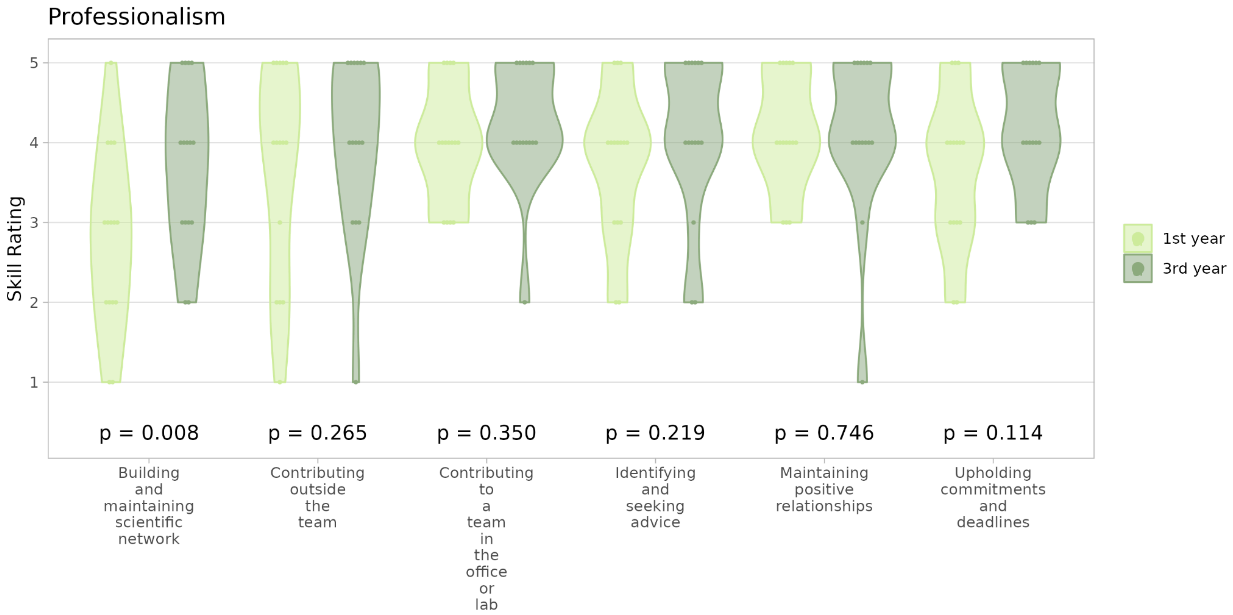
Professionalism
Skill Rating
5
4
3
2
1
p = 0.008
p = 0.265
p = 0.350
p = 0.219
p = 0.746
p = 0.114
Building and maintaining scientific network
Contributing outside the team
Contributing to a team in the office or lab
Identifying and seeking advice
Maintaining positive relationships
Upholding commitments and deadlines
1st year
3rd year

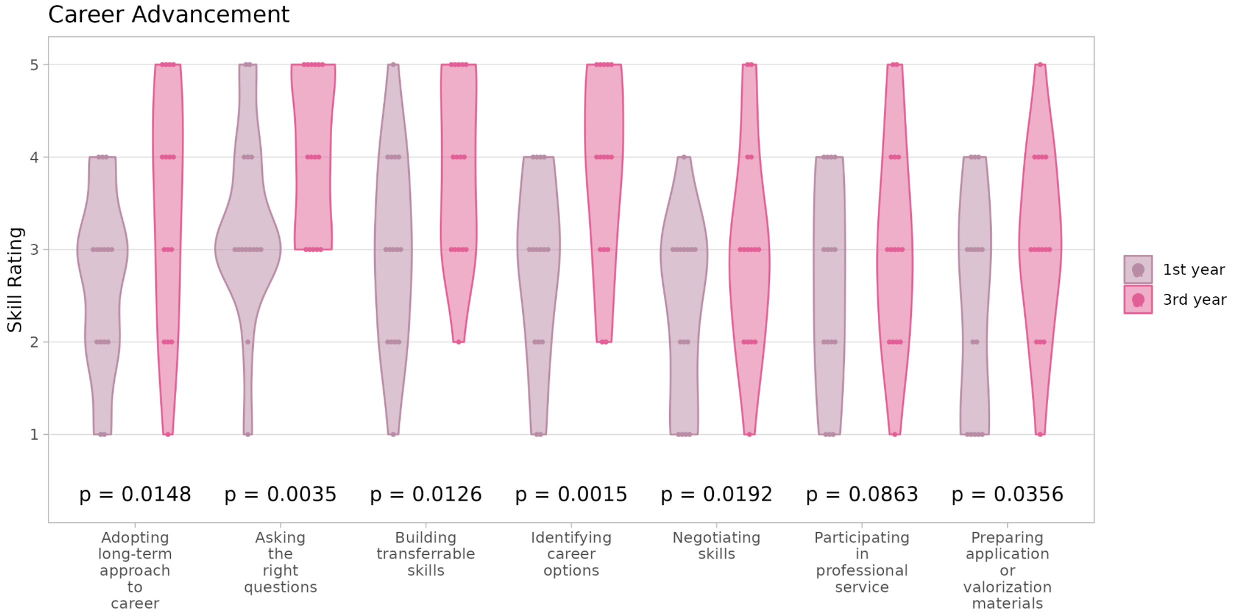
Career Advancement
Skill Rating
5
4
3
2
1
p = 0.0148
p = 0.0035
p = 0.0126
p = 0.0015
p = 0.0192
p = 0.0863
p = 0.0356
Adopting long-term approach to career
Asking the right questions
Building transferrable skills
Identifying career options
Negotiating skills
Participating in professional service
Preparing application or valorization materials
1st year
3rd year

Supplementary Figure 2: **Impact of TranSYS bootcamps (top) and secondments (bottom) on ESR growth - professionalism, management & leadership, career advancement**

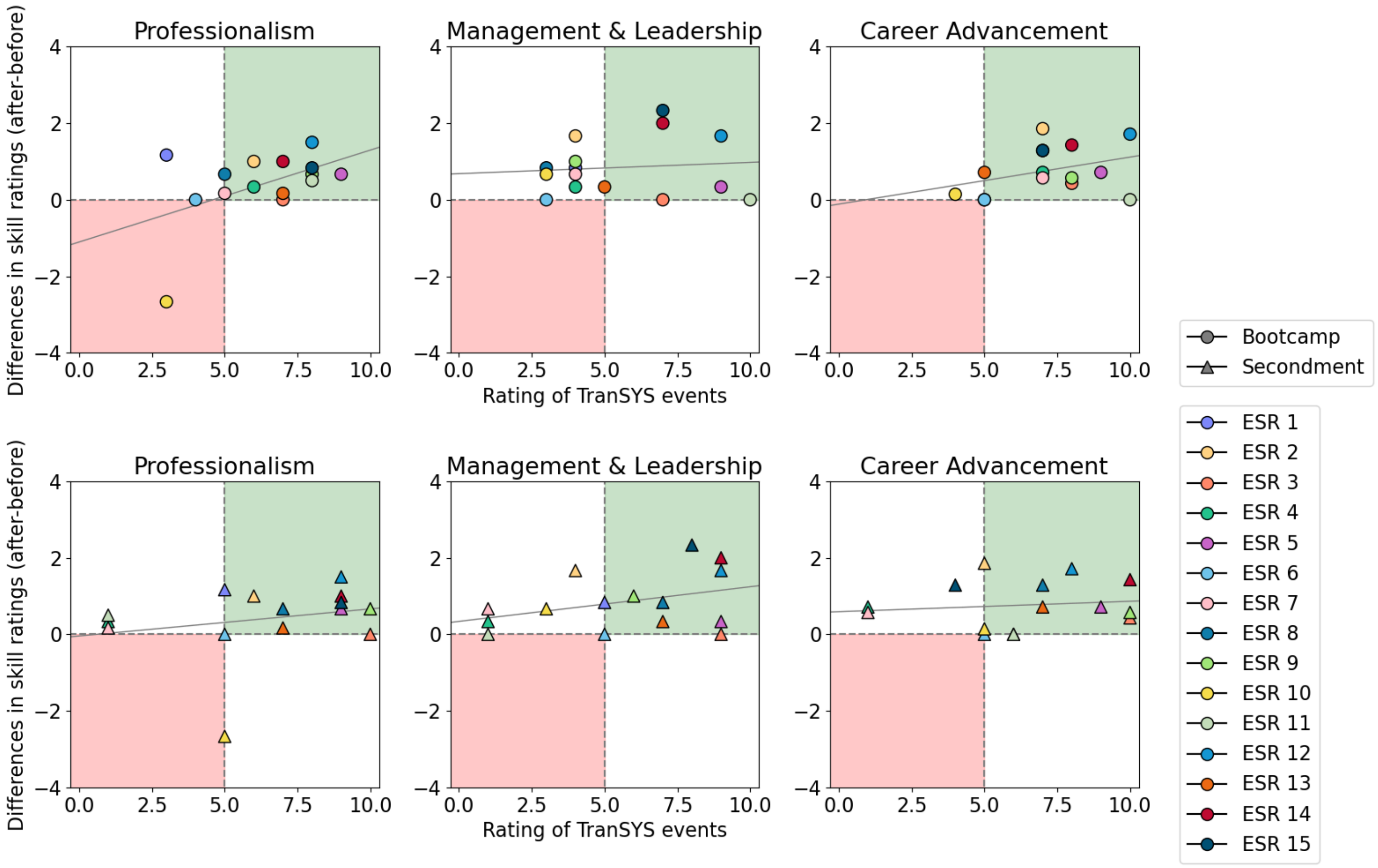

Supplement: Supplementary file 1 [file Data_Sheet_1.pdf]
